# The impact of microbes in the orchestration of plants’ resistance to biotic stress: a disease management approach

**DOI:** 10.1007/s00253-018-9433-3

**Published:** 2018-10-12

**Authors:** Matthew Chekwube Enebe, Olubukola Oluranti Babalola

**Affiliations:** Food Security and Safety Niche Area, Faculty of Natural and Agricultural Sciences, North-West University, Private Bag X2046, Mmabatho, 2735 South Africa

**Keywords:** Agriculture, Acquired systemic resistance, Induced systemic resistance, Plant immunity, Plant growth-promoting microbes, Plant pathogen

## Abstract

The struggle for survival is a natural and a continuous process. Microbes are struggling to survive by depending on plants for their nutrition while plants on the other hand are resisting the attack of microbes in order to survive. This interaction is a tug of war and the knowledge of microbe-plant relationship will enable farmers/agriculturists improve crop health, yield, sustain regular food supply, and minimize the use of agrochemicals such as fungicides and pesticides in the fight against plant pathogens. Although, these chemicals are capable of inhibiting pathogens, they also constitute an environmental hazard. However, certain microbes known as plant growth-promoting microbes (PGPM) aid in the sensitization and priming of the plant immune defense arsenal for it to conquer invading pathogens. PGPM perform this function by the production of elicitors such as volatile organic compounds, antimicrobials, and/or through competition. These elicitors are capable of inducing the expression of pathogenesis-related genes in plants through induced systemic resistance or acquired systemic resistance channels. This review discusses the current findings on the influence and participation of microbes in plants’ resistance to biotic stress and to suggest integrative approach as a better practice in disease management and control for the achievement of sustainable environment, agriculture, and increasing food production.

## Introduction

Defense is a strategy for survival, and for any organism to survive in this interdependent environmental ecosystem, it must defend itself or experience extinction. Every creature possesses one or more defense tools and plants are no exception. To sustain its health, vitality, and existence, plants must ward off and counteract the actions of their enemies (pathogens) through many different modes including the production of secondary metabolites known as phytoalexins or phytoanticipins (Khare et al. 2017).

**Fig. 1:**
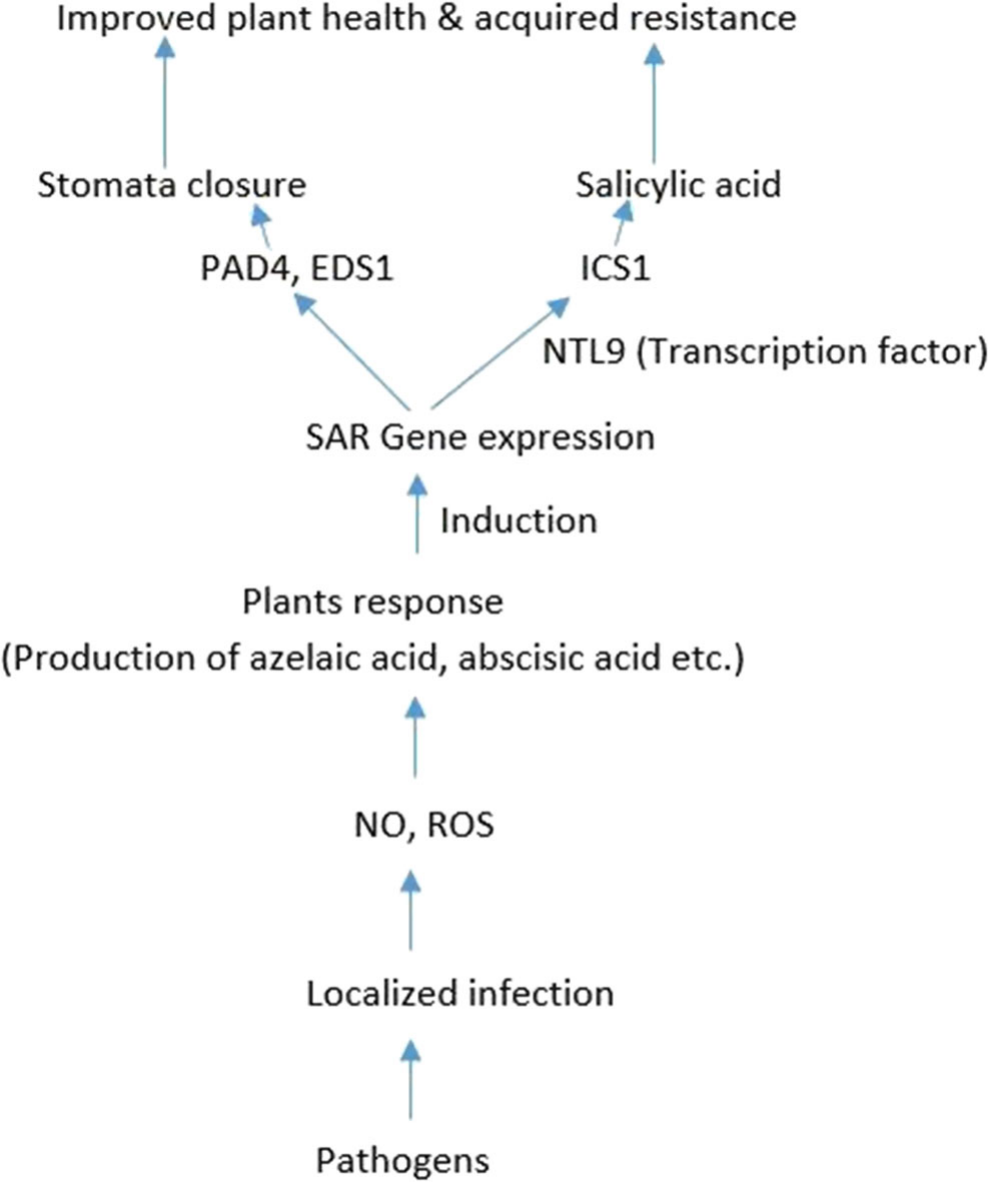
The impact of pathogen infection in the acquisition of systemic resistance in plant. *PAD4* phytoalexin deficient 4, *EDS1* enhanced disease susceptibility 1, *ICS1* isochorismate synthase 1, *NTL9* NTM1-LIKE9 transcriptional factor, *NO* nitric oxide, *ROS* reactive oxygen specie

The survival of plants depends on their ability to defend themselves through local and systemic responses with respect to an invasion or sensing of the presence of pathogens. These defense signals are triggered by microbes (Fig. 1) at the site of infection that leads to multiple protective responses against the invader and other unrelated pathogenic species (Pieterse et al. 2014). Biotic stress induces the production of oxygen-derived radicals such as H_2_O_2_ (hydrogen peroxide), superoxide molecules, hydroxyl, and/or oxygen radicals that are the first lines of defense for a stressed plant (Nanda et al. 2010). However, certain plant hormones (salicylic acid, jasmonic acid, ethylene) and substances like hydrogen peroxide and oxygen radicals are often implicated in the initiation and control of these phytodefense activities that trigger the production of phytoalexins, callose depositions, cell wall thickening/strengthening, metabolite production, and pathogenesis-related protein synthesis. Together, these responses intercept and inhibit the action of the invading pathogens (Vinale et al. 2008; Singh et al. 2016; Nie et al. 2017). These defense proteins (enzymes) are remarkable in the protection of the plant via the reaction processes they catalyze. The biosynthesis of phytoalexins and/or phenolic compounds as well as salicylic acid production is catalyzed by phenylalanine ammonia lyase. Polyphenol oxidase facilitates the redox reaction that converts polyphenol to quinone antimicrobial compounds (Gong et al. 2017). However, in the absence of biocontrol microbes, pest- or pathogen-challenged plants could produce in excess of phenylalanine ammonia lyase (PAL) (Fukasawa-Akada et al. 1996; Vanitha et al. 2009), beta 1,3-glucanase, chitinase (Mauch et al. 1988), peroxidase (Van Lelyveld and Brodrick 1975), superoxide dismutase (Lu et al. 2017), and peroxidase biomolecules (Hammerschmidt et al. 1982). Pathogen-infected plants produce many compounds including alkaloids, phenolics, glucosinolates, betanins, terpenoids, cyanogenic glucosides, etc. These compounds are produced by infected cells and surrounding tissues during and after the infection. These substances can prevent pathogens from further infection of the plant (Srikantaramas et al. 2008). These defense mechanisms could best be described as an intrinsic resistant strategy by plants to biotic stress.

In the presence of beneficial microbes, pathogen-stressed plants undergo a partial or complete reprogramming of their metabolic pathways involved in the defense signaling processes to activate appropriate pathogenesis-related pathways (Singh et al. 2016). These defense mechanisms are metabolically costly to the plant. The semi-intrinsic part of the plant defense process involves reprogramming of the defensive response in plants as engineered by the rhizosphere soil microbiome. These microbiome populations are attracted by plants. Plants play a central role in the selection, initiation, and recruitment of potential microbes that will form its rhizosphere microbiome through the type and nature of exudates it releases into the rhizosphere (Spence et al. 2014; Berendsen et al. 2012). These recruited microbes include some that are beneficial and others that are antagonistic, will interact with the plant receptors, and prime their immunity. The defense immune priming in plants is initiated once the microbial extracellular structures and molecules such as exopolysaccharide, proteins, flagellins, etc., come in contact with the cell receptors on the surfaces of plant. Also, local infection of plants by pathogens as well as herbivore attack will result in a structural and functional damage of the affected part. This disruption in the structural and functional network perhaps will initiate a signal transduction from the local site of attack to other parts of the plant for proper immune sensitization. This process is mediated by the amino acid glutamate. Glutamate receptor-like family bearing charged groups and ions will pick up these signals associated with tissue damage and hence induce the accumulation of calcium ions within the plant cells. The accumulated ions will relay the impulse to distant organs responsible for the activation of the defense response genes. Therefore, glutamate triggers long-distance, calcium-based plant defense signaling (Toyota et al. 2018). In other words, this initiates the activation of a cascade of defense genes to produce reactive oxygen molecules, superoxide dismutase, peroxidase, and a host of other biomolecules (Luiz et al. 2015). These chemical substances work both within and outside plants to bring forth desired inhibitory effects on the pathogen. Priming of defense genes in plants as a result of inducers (microbes) or elicitors is termed induced systemic resistance (Stangarlin et al. 2011).

Beneficial nonpathogenic microbes interact directly with the pathogens by secreting chemical metabolites that will suppress their growth and/or render them avirulent, thereby protecting the host plant (Dey et al. 2014). This mechanism is a direct plant pathogen-assisted control by rhizomicrobes. A gram-positive microbe *Micromonospora* obtained from the root nodules of legumes has exhibited a direct biocontrol by inhibiting the growth of many fungal pathogens. This microbe also induces jasmonic acid signaling defense in tomato plants exposed to the fungus *Botrytis cinerea* (Martinez-Hidalgo et al. 2015).

Over the years, the use of agrochemicals (fungicides and pesticides) to control pathogens of crops has been found to constitute an environmental hazard and causes bioaccumulation of toxic substances in the food chain. This necessitates the need for adoption of an eco-friendly alternative in solving the problem and in sustaining the environment. The use of plant growth promoting microbes has been shown to be a good option in the fight against pathogen invasion of crops (Ashwin et al. 2017; Bohm et al. 2014). With the need to boost yield and health of crops and to minimize the involvement of agrochemicals in crop disease management, identification of viable biocontrol agents as well as uncovering mechanisms and mediators of plants’ resistance to biotic stress (as seen in the glutamate-induced long-distance defense signaling above) is paramount.

## Microbial induction of systemic resistance in plant to biotic stress

The production of chemical substances and their transportation through protein channels to the site of infection and to other parts of the plant (Khare et al. 2017) undergoing a stressful condition is a direct approach to the sustenance of plant’s health and vitality. The plant *Arabidopsis thaliana* possess high numbers of transport proteins (ABCG34) and has the ability to resist invasion of the fungus (*Alternaria brassicicola*) by producing and transporting the fungicidal substance camelexin to the surface of the plant leaves. The presence of this fungus stimulated the production of metabolites as well as the expression of genes (*AtABCG34*) responsible for the production of the transport protein (Khare et al. 2017). *Nicotiana tabacum* producing sclareol (diterpene alcohol) enhances the plant defenses against invading pathogens (Crouzet et al., 2013).

The biosynthesis/production of jasmonic acid within plants as a result of physiological defense impact-response of plants to pathogen invasion/attack contributes to the growth of the plant and also inhibits pathogenic infection reoccurrence. Jasmonate-induced oxygenase inactivates the jasmonic acid activity by hydroxylation reaction to bring its activities to normal as seen in *Arabidopsis*. An Arabidopsis mutant possessing a dysfunctional gene responsible for production of the oxygenase enzyme produced an overproduction of jasmonic acid that resulted in the inhibition of plant growth but increased the plant resistance to invasive pathogens (Caarls et al. 2017). In the presence of pathogens, salicylic acid is produced through the transcription and induction of the major synthetic gene known as isochorismate synthase 1 which is activated by the transcriptional factors (NTM1 – LIKE 9 and CCA1 HIKING EXPEDITION). As seen in the induction of acquired immunity in plants, salicylic acid not only serves as a hormone but is also responsible for local and systemic resistance of plants to pathogens. It facilitates the production of plant proteins that are microbiocidal in nature. It is involved in the stomatal regulation/behavior in the presence of pathogenic microbes on the phylloplane and enhances efficient closure of this pathway against the entrance of the pathogen into the plant tissue. To perform this regulatory role, plants engage their surface receptors (flagellin sensing 2) that sense the presence of microbe-associated proteins such as flagellin which triggers the closure of the stomata and prevents the entrance of the pathogen into the plant (Zheng et al. 2015; Zeng and He 2010).

The rhizobacteria flora native to the soil has significantly reduced the incidence of disease and death of tobacco (*Nicotiana attenuata*) inflicted by *Fusarium* or *Alternaria* compared to plants infected growing in a fungi-infested agricultural soil (Santhanam et al. 2015).

**Fig. 2:**
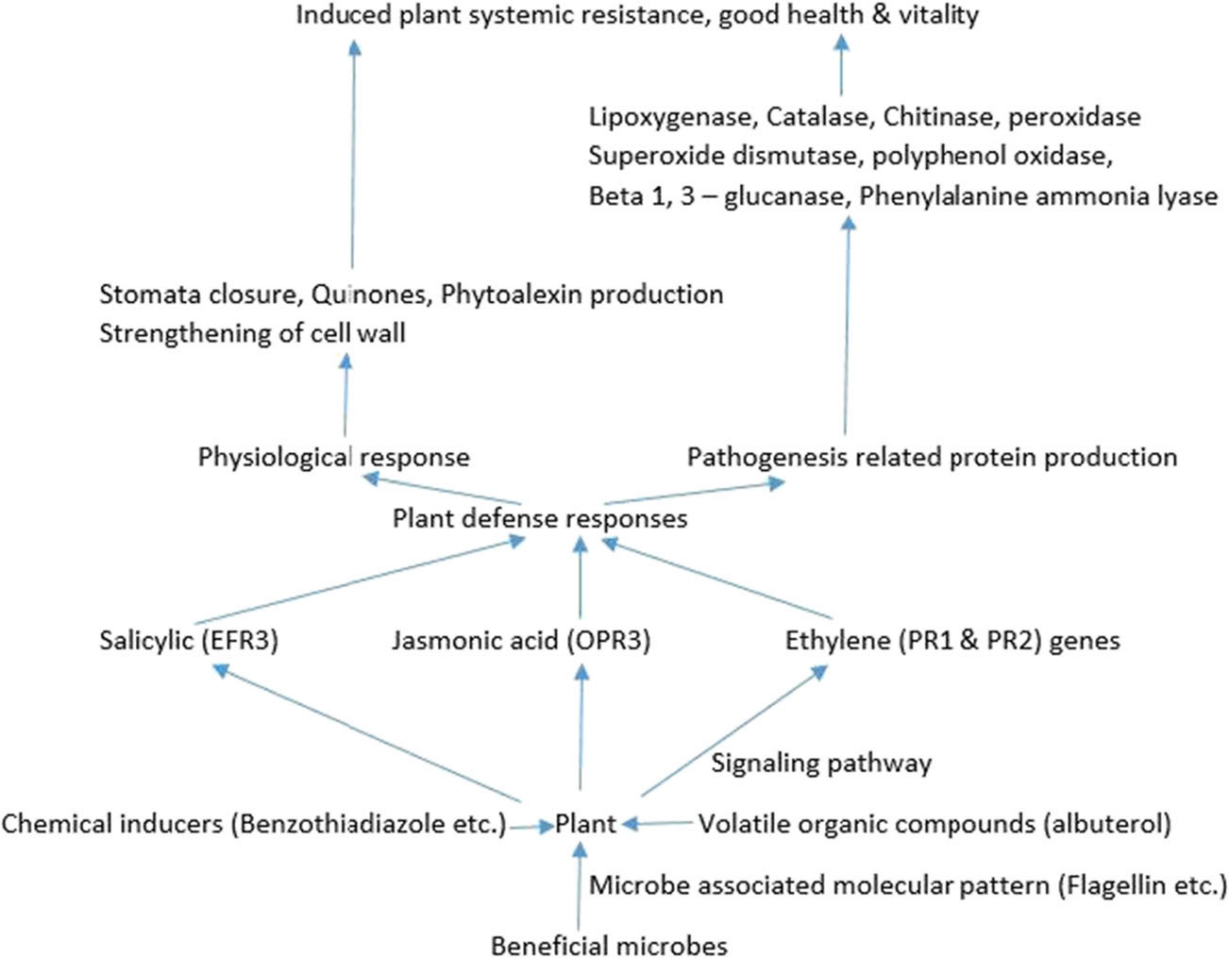
The interrelatedness of beneficial microbes, chemical inducers, and elicitors in the induction of systemic resistance in plants. *EFR3* ethylene response factor, *OPR3* jasmonic acid signaling gene, *PR1* pathogenesis-related protein, *PR2* beta 1,3 – glucanase

The application of exopolysaccharides produced by *Lactobacillus plantarium* on tomato plant elicited/induced the expression of defensive genes as observed with increased expression of the intracellular defense enzymes: catalase (CAT), polyphenoloxidase (PPO), superoxide dismutase (SOD), and hydrogen peroxide (H_2_O_2_). These substances (Fig. 2) enhanced plant resistance to the destructive pathogen *Xanthomonas gardneri*, the agent that causes bacterial spot disease on tomato leaves. Exopolysaccharide treatment influenced the lowering of water movement and escape from the leaves’ stomata pores by 36% (Blainski et al. 2018). Also, a protein molecule (*Colletotrichum falcatum* plant defense-inducing protein 1) produced by the fungus *C. falcatum* (a pathogen of sugar cane) was able to prime the expression of the defense machinery of sugar cane. This induction of the defense genes lead to the inhibition of the fungus-associated cellular lesion on the treated sugar cane plant that was challenged with the pathogen *C. falcatum*. Noticed also was stimulation of the plant’s production of hydrogen peroxide and deposition of callose on the affected plant parts (Ashwin et al. 2018).

A tomato rhizosphere associated bacteria—*Pseudomonas* sp.—capable of producing the antimicrobial substance phenazine enhanced the intrinsic resistance of the tomato root and shoot to the attack of pathogens. It stimulated the intracellular accumulation of organic compounds (phenolics, lipoxygenase, and jasmonic acid) in the treated plant and provided protection against a wide range of pathogenic microbes (fungi, bacteria, and/or viruses) (Hariprasad et al. 2013). The gene products responsible for the control of pathogenesis-related genes has shown that *Lox3-4*, *ZmLox3* (lipoxygenase gene), negatively control the expression of genes capable of promoting systemic resistance in plant. But the disruption of this gene will generate a Lox3-4 mutant in maize plant. This significantly increased the leaves’ systemic resistance to the pathogen *Colletotrichum graminicola*. In the absence of this negative control gene, the pathogenesis genes were constitutively expressed to sustain the resistance of the plant to pathogens (Constantino et al. 2013).

In the same vein, soybean treated with the bacterium *Bacillus* sp. CHEP5 remarkably stimulated the intrinsic resistance of the plant to the fungus *Cercospora sojina* Hara, an agent that causes frogeye leaf spot (FLS) disease in soybean plants. It equally empowered the plant to express defense genes responsible for jasmonic acid synthesis (Tonelli and Fabra 2014). Although bacteria involvement in induction of systemic resistance has been known, the issue is on whether this is scalable and feasible for a field crop disease control, with respect to the dynamic climate condition and varying agricultural practices and soil management. Mycorrhizal fungi are equally implicated in the priming process of plant protective genes. They do this by root colonization and through the signaling pathway, prime jasmonic and salicylic acid synthetic genes (OPR3 and PR1). These primed genes will then boost tomato resistance (for instance) to *Alternaria alternata* infection. Also primed were the genes involved in the biosynthesis of enzymes such as lipoxygenase (LOX) and phenyl ammonia lyase (PAL) (Nair et al. 2014).

From a different perspective, bacterial involvement in systemic resistance of barley plants could proceed through an indirect priming of the non-expressor pathogenesis-related genes by activating the transcriptional factors (WRKY and ethylene responsive factor) which then resulted in transcriptional reprogramming of the plant for effective induction of the expression of pathogenesis-related genes. Also, exogenous application of salicylic acid (Table 1) could play a major role in plant immunity, as well as jasmonic acid methyl ester and/or abscisic acid which induce the systemic resistance of plant to *Xanthomonas translucens* pathovar *cerecilis* infection (Dey et al. 2014). Among the myriad of microbes found at the rhizosphere of maize plant, *Pseudomonas putida* KT2440 is an excellent root-associated bacterium and could tolerate the toxicity of maize root exudates that are inhibitory to other microbes. The mutual affinity between maize and pseudomonas enabled the bacterial presence to trigger the expression of jasmonic and abscisic acid production in the early phase of the plant relationship with the bacteria. This relationship manifested in the suppression of the gene expression of salicylic acid. However, the plant response to the bacteria presence gradually fades away as the plant begins to adjust and accommodate the presence of the associative partner. This microbe equally induced systemic resistance of maize to the infection of the fungus *Colletotrichum graminicola* which causes leaf necrosis (Planchamp et al. 2015). To achieve this immune enhancement of plants by microbial influence, effective communication is a prerequisite.

**Table 1:** Microbial elicitors that induce systemic resistance in plants

Microbes	Organic substance produced	Phytopathogens	Plants	References
*Bacillus subtilis* 985, *Bacillus amyloliquefaciens* 5499	Surfactin	*Botrytis cinerea*	Tobacco	Cawoy et al. (2014)
*Escherichia coli* (recombinant)	PevD1 protein	*Verticillium dahliae*	Cotton	Bu et al. (2014)
*Cladosporium* sp., *Ampelomyces* sp.	m-Cresol methyl benzoate	*Pseudomonas syringae* (pv. tomato DC3000)	*Arabidopsis thaliana*	Naznin et al. (2014)
*Bacillus subtilis*	Culture supernatant	*Meloidogyne incognita*	Tomato	Adam et al. (2014)
*Phytophthora parasitica* OPEL protein from Recombinant *E. coli*	OPEL protein	*Tobacco mosaic virus*, *Ralstonia solanacearum*, *Phytophthora parasitica*	*Nicotiana tabacum* (cv. Samsun NN)	Chang et al. (2014)
*Bacillus subtilis*	Surfactin, mycosubtilin	*Botrytis cinerea*	Grapevine	Farace et al. (2014)
*Pseudomonas fluorescens* RRLJ134, *Pseudomonas aeruginosa* RRLJ04	Phenazine analogues	*Fomes lamoensis*, *Ustulina zonata*	Tea	Mishra et al. (2014)
*Trichoderma virens*, *Trichoderma atroviride*	SM1 (small protein1) and EPl1 proteins (eliciting plant response-like protein)	*Alternaria solani*, *Botrytis cinerea*, *Pseudomonas syringae* pv. tomato (Pst DC3000)	Tomato	Salas-Marina et al. (2015)
*Bacillus* sp. SJ	Volatile compounds	*Rhizoctonia solani*, *Phytophthora nicotianae*	Tobacco	Kim et al. (2015)
*Bacillus fortis* IAGS 162	Phenylacetic acid	*Fusarium oxysporum* f.sp. *lycopersici*	Tomato	Akram et al. (2016)
*Bacillus subtilis* DZSY21	Lipopeptides	*Bipolaris maydis*	Maize	Ding et al. (2017)
*Pseudomonas aeruginosa* PM12	3-Hydroxy-5-methoxy benzene methanol (HMB)	*Fusarium oxysporum*	Tomato	Fatima and Anjum (2017)
*Bacillus subtilis* SYST2	Albuterol, 1,3-propanediol	*Ralstonia solanacearum* TBBS1	Tobacco	Tahir et al. (2017)
*Pseudomonas protegens* CHAO	Orfamide A	*Cochlibolus miyabeanus*	Rice	Ma et al. (2017)
*Bacillus amyloliquefaciens* (UCMB5113)	Fengycins	*Alternaria brassicicola*	*Arabidopsis thaliana*	Asari et al. (2017)
*Saccharothrix yanglingensis* (Hhs.015)	BAR11 protein	*Pseudomonas syringae* pv. tomato DC3000	*Arabidopsis thaliana*	Zhang et al. (2018)
*Bacillus amyloliquefaciens*, *Bacillus subtilis*	Iturin A, Fengycin, Bacillomycin	*Fusarium moniliforme*	Maize	Gond et al. (2015)

This communication is mediated by exchange of signaling molecules or proteins at the rhizosphere (Babalola 2014). *Trichoderma virens* have been found to excrete small, secreted cysteine proteins (SSCPs) which enhance the symbiosis between the microbe and the plant. These molecules perform a positive effector role in the sustenance of plant’s defense to parasites as well as pathogens and in promoting the establishment of a symbiotic relationship between the plant and fungi. They are equally involved in the control of induced systemic resistance of plant by *Trichoderma virens*. These beneficial rhizosphere interactions empower the plant to tolerate and/or resist pathogen infection such as resistance of maize to *Cochliobolus heterostrophus* (Lamdan et al. 2015).

Co-inoculation of plant growth promoting rhizobacteria (*Pseudomonas* sp. R41805) with mycorrhizal fungi (*Rhizophagus irregularis* MUCL 41833) stimulated the activation of systemic defense genes of the potato plant against *Rhizoctonia solani* infection through the priming of the ethylene resistance network. This is an indirect approach to biocontrol of plant pathogens (Velivelli et al. 2015). In a direct biocontrol, *Pseudomonas fluorescens* LBUM223 possessing the intrinsic ability to produce Phenazine – 1 – carboxylic acid was found to control *Streptomyces* sp. involvement in the infection of potato by negatively regulating the gene (*txtA*) expression of the *Streptomyces*. This virulence and pathogenicity gene in *Streptomyces* responsible for thaxtomic production is involved in scab disease development in potato (Arseneault et al. 2015). The influence of root-associated *Pseudomonas fluorescens* PTA-CT2 was felt in grapevine both local and systemic, as the bacteria induce systemic resistance of the plant to the pathogen *B. cinerea*. This point source as well as systemic (in roots and leaves) influence could be attributed to the transfer of metabolites from the root to the upper chamber of the plant. It also induced the expression of phytoalexin and glutathione 3-transferase with a marked decrease in the expression of hypersensitive related genes. Cell death was equally observed in the plant roots (Gruau et al. 2015). Indeed, *Phytophthora cactorum*, pathogen of the Korean ginseng plant that caused the dreaded root disease, was efficiently controlled by a plant growth promoting rhizobacteria (*Bacillus amyloliquefaciens* strain HK34) which induced systemic resistance in ginseng plant via inducing the expression of pathogenesis-related genes in the treated plant (Lee et al. 2015).

The immune response of plants to microbes could either follow one of the two main routes: SAR—systemic acquired resistance and/or ISR—induced systemic resistance. These two routes will arrive at the same point of boosting the plant immunity. Induced systemic resistance involves the activation of the plant immunity by plant interaction with beneficial nonpathogenic microbes such as the plant growth-promoting rhizomicrobes. These microbes stimulate the activation of plant immunity via its contact with the plant receptor responsible for sensing the microbe-associated molecular pattern of the microbe (Zamioudis and Pieterse 2012; Pieterse et al. 2012). Moreover, the mechanisms and signaling processes involved in microbial induction of systemic resistance have been well reviewed by Shine et al. 2018.

The two main signaling pathways (salicylic acid and jasmonic acid/ethylene) were primed simultaneously by *Bacillus cereus* AR156 in *A. thaliana* plant. The activation of these pathways/defense genes gives rise to improved plant immunity and resistance to pathogenic microbial infection. These genes are found to be controlled by two transcriptional factors (WRKY11 and WRKY70). The presence of *B*. *cereus* has a positive stimulatory effect on the activity of WRKY70 but negatively suppresses WRKY11 in the plant. Nevertheless, transcriptional factors enable proper transcription of DNA and contribute greatly in the process of *B. cereus* induction of systemic resistance in plant. And the microorganism equally has the tendency to activate both salicylic acid and jasmonic acid signaling pathways simultaneously in the green vegetative plant (Jiang et al. 2015).

In line with the forgoing, mycorrhizal fungi a beneficial symbiotic microbe facilitated the induction of systemic resistance in tomato plant. This fungus on its own does not induce the priming of the pathogenesis-related genes but only do so in the presence of a pathogen. The arbuscular mycorrhizal fungus (*Funneliformis mosseae*) facilitated tomato resistance to *Alternaria solani sorauer* infection. The organism lead to an increase in beta 1,3-glucanase, chitinase, PAL, and Lox in the tomato leaves when inoculated with the microbial pathogen. Therefore, in the presence of a pathogen, arbuscular mycorrhizal fungi pre-inoculated-tomato plants have the highest defensive gene response involved in pathogenesis (namely PR1—pathogenesis-related protein, PR2—beta 1,3-glucanase, and PR3—chitinase) and defense-related genes (Lox, allene oxide cyclase (AOC), PAL) in the leaves of the tomato plant (Song et al. 2015).

A nonpathogenic bacteria, *Rhizobium radiobacter*, a close cousin of *Agrobacterium tumifaciens* (now called *R. radiobacter* biovar 1 strain C58), has the ability to activate/induce the expression of plant defense genes and boost plant immunity through jasmonic acid signaling pathway induction. This was observed in *Arabidopsis* challenged with the microbe *Pseudomonas syringae* (pv. tomato DC3000). Obviously, similar effects were observed in wheat plants challenged with *Xanthomonas translucens* (pv. Translucens*)* (xtt) (Glaeser et al. 2016). The contributions of beneficial microbes in food production through the induction of systemic resistance of plants to pathogens cannot be overemphasized. This is clearly observed in the influence of *B. cereus* C1L to increase the vegetative growth of maize plant and improve its resistance/tolerance to pathogenic fungal-induced disease condition (southern leaf blight that is caused by *Cochliobolus heterostrophus*). This alternative and eco-friendly approach to biocontrol and promotion of plant immunity through the application of a microorganism that primes plant defensive genes has a significant substituting effect to farmers’ dependence on fungicides (Dithiocarbamate and mancozeb) in the control of fungi infection of plants. Unfortunately, these fungicides are capable of causing neurological disease/disorder in human beings (Parkinson’s) and necessitate the use of eco-friendly microbes as a substitute (Lai et al. 2016; Ferraz et al. 1988; Meco et al. 1994).

A look at induced systemic resistance from another dimension suggested that yeast (*Pseudozyma churashimaensis* strain RGJ1) isolated from a pepper leaf surface exerted a protective role on the plant against the viral infections caused by the cucumber mosaic virus (CMV), pepper mottle virus (PMV), pepper mild mottle virus (PMMV), and broad bean wilt virus (BBWV) and against the bacterial pathogen *Xanthomonas axonopodis*. The yeast was able to boost plant immunity through the induction of plant pathogenesis-related genes involved in the salicylic/jasmonic acid signaling pathway (CaPR4) and ethylene (CaPR5) signaling pathway (Lee et al. 2017). Whenever microbes succeed in infecting a plant, the cellular level of hydrogen peroxide will increase as well as the deposition of callose in the affected plant part as a first line of defense response by the plant (Nie et al. 2017). However, treatment of *A. thaliana* with *B. cereus* (AR156) promoted the plant immunity against *B. cinerea*. The protection involved many phases of induced systemic responses that encompass the expression of protein (PR1), H_2_O_2_, and deposition of callose. These physiological activities were observed more in *Arabidopsis* pretreated with *B. cereus* and later challenged with the *B. cinerea* pathogen. Induced resistance is as a result of the activation of the jasmonic acid/ethylene dependent signaling pathway and NPR1 (non-expressor of PR1) signaling pathway (Nie et al. 2017).

Synergy is often the best approach to achieving excellent results in any biological system. The co-inoculation of *Bacillus* sp. (CHEP5) and *Bradyrhizobium japonicum* (E109) enhanced the induction of soybean resistance to *C. sojina* infection. This agent causes frog leaf spot disease in soybean plant. The inductive capacity of these microbes could be ascribed to their ability to form a biofilm when grown together as well as priming of the plant defense immune system (Tonelli et al. 2017).

The microbial approach to pathogen infestation control through induction of systemic resistance in plants can be perpetuated by antagonistic microbe (*Pseudomonas* sp. S2 and S4) not only enhanced plant growth but also reduced and controlled the epiphyte microbe *Salmonella enterica*, the agent of food crop associated salmonellosis in tomato, spinach, and lettuce. Inoculation of this microbe on the root of vegetable had an indirect biocontrol effect on the phylloplane microbial pathogen through the induction of systemic resistance in the inoculated plant (Hsu and Micallef 2017). The effect of *Burkholderia phytofirmans* (PsJN), a plant useful endophyte, was observed in the suppression of the pathogen *P. syringae* (pv. tomato DC3000) against its infection on *A. thaliana*. The presence of this microbe (*B. phytofirmans*) on the roots of *Arabidopsis* caused the expression of the salicylic acid defense gene (PR1) and the expression of PDF1.2 (a jasmonic acid and ethylene regulated gene) which fortified the immune strength of the plant against infection (Su et al. 2017a). A similar event was noticed in a cucumber plant pretreated with the fungus *Trichoderma atroviride* (TRS25) which induced resistance in the plant against *R. solani* infection. The pretreatment exercise that resulted in *Rhizoctonia* inhibition was a result of increased treatment activation of plant defense enzymes—guaiacol peroxidase (GPX), syringaldazine peroxidase (SPX), phenylalanine ammonia lyase (PAL), and polyphenol oxidase (PPO). Also increased was the concentration of intracellular phenolic compound, hydrogen peroxide, and a corresponding decrease in thiobarbituric acid concentration. The fungus equally promoted the accumulation of derivatives of salicylic acid— methyl salicylate (MeSA), ethylhexyl salicylate (EHS), salicylic acid glucosylated conjugates (SAGC), beta cyclocitral, and volatile organic compound (VOC)—2,3 hexanal, 2,3-hexenol, and E-2-hexenal. These compounds particularly the volatile organic compound contributed greatly in the fungal induction of salicylic acid defense genes (PR1 and PR5) involved in the plant’s systemic acquired resistance (Nawrocka et al. 2018).

The influence of the BjNPR1 protein was found to contribute significantly to the *Brassica juncea* resistance to *Alternaria brassicae* and *Erysiphe cruciferarum* infection in a transgenic Brassica plant overexpressing the *BjNPR1* gene (Ali et al., 2017). The bacterium *Bacillus* sp. capable of inducing the production of antioxidant defense enzymes (superoxide dismutase, peroxidase, polyphenol oxidase, phenylalanine ammonia lyase) in rice plant strengthened its resistance to *Pyricularia oryzae* infection (Rais et al. 2017).

Although beneficial microbes are inducing systemic resistance in plants against microbial infection, yet the struggle for dominance and survival allows some pathogens to constantly devise a means to avert the inhibitory effect of plant immune components. These microbes end up producing HC-toxin (a histone deacetylase inhibitor) which reprogram the transcriptional response of plant to microbial infection and succeed in making the plants’ immune defenses ineffective. This HC-toxin is produced by the pathogen (*Cochliobolus carbonum* race 1). The toxic substance increased the virulence and infection capacity of the microbe and also changed the acetylation of proteins in maize plant (Walley et al. 2018). The big problem is what are the chances that this HC-toxin gene will not be transferred to other microbes that are beneficial to plant? If this toxin-producing gene were to be transferred either horizontally or vertically, this will render the farmers’ effort in crop production futile. For this reason, more studies should be channeled toward the identification of potential biocontrol agents that can antagonize *C. carbonum* without picking up the toxin gene from it. At present, there is very little work done in this area to help solve the problem. A good strategy will always involve cooperation and/or division of labor. Co-inoculation of peanut (groundnut) plants with *Bacillus* sp. (CHEP5 specie) and *Bradyrhizobium* sp. (SEMIA6144 specie) remarkably protected the plants from the attack of the *Sclerotium rolfsii* (the agent that cause plant stem wilt disease) and increased the plant immunity together with the yield of the treated plant (Figueredo et al. 2017). Also, *Bacillus pumilus* and *Paenibacillus* sp. are able to secret volatile compounds (2,5 dimethyl pyrazine and 1-octen-3-ol) inhibited the proliferation of the fungus (*Phaeomoniella chlamydospora*, the agent that causes grapevine trunk disease). These microbes induced the expression/activation of the pathogenesis-related genes and callose synthase-genes in the plant. Therefore, production of antimicrobial/antagonistic substances take priority in the control of fungus which is followed by induction and/or priming of systemic resistance in the plant (Haidar et al. 2016).

## The dual role of an effective microbe

An effective microbe has a dual role—disease control and promotion of plant growth. This attribute is found with *Rhodopseudomonas palustris* GJ-22. These photosynthetic bacteria not only promote *Nicotiana benthamiana* (tobacco) growth by producing indole acetic acid and 5-aminolevulinic acid but also improved the plants’ resistance against tobacco mosaic viral infection through priming of pathogenesis-related genes (Su et al. 2017b). The dual role of the bacterium *Azospirillum* sp. B510 enable tomato plant to grow and be protected against infection by *P. syringae* (pv. Tomato) as well as *B. cinerea* which causes leaf spot and gray mold in the plant. *Azospirillum* enhances the immunity of the treated plant in a non-acquired systemic resistance manner (Fujita et al. 2017).

The question is can the use of these microbes be effective in the field? Is it scalable? If it is scalable, what is the probability that these biocontrol microbes will tolerate the stiff competition at the rhizosphere and adapt to the environmental condition? Would their competitive advantage (if any) enable them carryout their biocontrol activities in the soil? Many a time, an effective microbe at the laboratory or controlled laboratory experiment is usually a failure or ineffective under field condition.

On the other hand, root-associated beneficial microbes (*Pseudomonas simiae* WCS417) capable of inducing plant systemic resistance and also boost its growth are able to perform this function by suppressing quite a number of plant’s responses arising from its association with microbes. This is to enable it to interact mutually with the plant root without being interfered by the plant immune response at the root region (rhizosphere). The flagellin influenced the transcriptional activity of the plant. Microbial flagellin from live and dead microbes can equally elicit/trigger plant immune response (Stringlis et al. 2018). Microbes have the tendency to trigger plant immunity as well as promote its growth without anyone of these activities interfering with another (Huot et al. 2014). These unique characteristics of beneficial microbes could be exploited in disease control and management. Certain proteins are essential in the induction and expression of pathogenesis genes for a sustainable plant immunity. The protein of importance is NPR1 (known as non-expressor of pathogen-related gene1). They are transcriptional cofactor protein molecules that upon binding to the transcription factor (TGA) can enhance the transcription of salicylic acid pathogenesis genes (Tada et al. 2008; Cao et al. 1994).

## Biocontrol microbes in fruit preservation

Microbes have proven to be good candidates in the control of fruit spoilage organisms and hence useful in fruit preservation. For instance, *Cryptococcus laurentii*, a yeast capable of biocontrol of pathogen involved in postharvest fruit and vegetable spoilage, has encouraged the activation/priming of defense-related genes (salicylic and jasmonic acid signal pathways) and the expression of pathogenesis-related protein genes that together made cherry tomato resistant to *B. cinerea* and *A. alternata* infection of the fruit (Lai et al. 2018; Wei et al. 2016). Fruit preservation could be approached microbiologically by the application of antagonistic microbes that will prime the expression of pathogenesis-related genes in the fruit, raise the fruit immunity, and enable it to resist the infective action of the fruit spoilage organisms. Treatment of tomato with *Clonostachys rosea* excellently inhibited the pathogenic actions of *B. cinerea* on the fruit. *C. rosea* induced systemic resistance condition in the fruit as observed with the elevated level of indole acetic acid (IAA), salicylic acid (SA), nitric oxide, phenylalanine ammonia lyase (PAL), and polyphenol oxidase (PPO) and decreased the concentration of catalase (CAT) and abscisic acid (ABA) (Gong et al. 2017). However, utilization of microbes in the control of fruit spoilage organisms for an increase in fruits’ shelf life has raised quite a number of issues. What is the likelihood that these microbes will not constitute an environmental hazard as well as posse health-related challenges when used in fruit preservation? One of the major challenges that could limit the wider adoption of this phytopathogen control method is the possibility of microbial gene exchange in the environment. This requires that caution be applied to avoid pickup and transfer of virulent genes. Although this fruit preservation method is effective, the fact remains that until these issues are sorted out, using this method of fruit preservation in a commercial scale is full of risk.

## Systemic acquired resistance in plants: A second alternative

Systemic acquired immunity usually occurs when a necrophilic/necrotizing pathogen (i.e., pathogens that cause cell death upon infection of a living cell/tissue) attack a plant, leading to the priming of pathogenesis-related genes responsible for this immune response to be activated. Systemic acquired resistance can as well be described as a wide-spectrum disease resistance of plant following a localized infection that transmit protection/immunity to secondary infection of the same or to a different microbial pathogen in an uninfected part of the plant. It is found that NO (nitric oxide) and O_2_ radical species are implicated in the induction/activation of systemic acquired resistance in plant via cleaving the C9 double bond of C18 unsaturated fatty acid whose product is azelaic acid (the inducer of systemic acquired resistance) (El-Shetehy et al. 2015). Also, the role of abscisic acid in the modulation of salicylic acid biosynthesis during tomato acquired systemic resistance is well documented by Kusajima et al. (2017).

At the gene level, induction of salicylic acid production during systemic acquired resistance by plant involves the transcription of the gene ICS1 (isochorismate synthase 1). Activation of this gene upon pathogen attack is controlled by the transcriptional factors NTL9 (NTM1-LIKE9) and CHE (CCA1 HIKING EXPEDITION) responsible for priming the ICS1 gene for immune responses to specific pathogen. Transcriptional factor NTL9 not only induces the expression of ICS1 but also the expression of PAD4 (phytoalexin deficient 4) and EDS1 (enhanced disease susceptibility 1) located within the guard cells of the leaves stomata where sensitization (Fig. 1) and expression of the gene help to boost the immunity and closure behavior of the stomata in response to pathogen presence (Zheng et al. 2015).

## Plant influence in soil immunity buildup

Many soils harbor a consortium of both beneficial and pathogenic soilborne microbes but naturally through root exudate secretions, microbes could be either supported or starved depending on their ability to metabolize the exudates. As the number one major primary producers in the ecosystem, photosynthesis is the only means by which plants synthesize and supply labile carbon as well as poly-sugars to the soil-dwelling microbes. This gives them the influence over which organism should prevail and which to suppress through starvation. Aside from photosynthates injection into the soil, plants also introduce a number of antimicrobial substances that could inhibit the growth of certain microbes.

The actual plant protection and disease suppression inherent in the soil could equally be attributed to the rich diversity, structure, and function of viable microbes attracted and supported by the plants. These microbes will proliferate and out-compete the pathogenic microbes or may secrete antimicrobials into the soil to outwit their competitors, thereby indirectly making the soil healthy for crop production.

However, microbes have been implicated in the induction of plants’ immune responses and protection from invasive pathogens. Yet the choice of which microbe to invite, support, and sustain is entirely dependent on the plants. Plants carry out these roles through their rhizosphere effects. This type of soil sustains the health of plants in spite of the presence or absence of soilborne pathogens. This “immune fortified” soil often occurs with agricultural practice of continuous cropping system involving planting the same crop such as wheat, sugar beet etc., in the same farmland till the soil enters a disease-suppressive mode (Raaijmakers and Mazzola 2016). *A. thaliana* attracted *Xanthomonas* sp. (WCS2014-23), *Stenotrophomonas* sp. (WCS2014-113), and *Microbacterium* sp. (WCS2014-259) in a defense against the attack of the pathogen *Hyaloperonospora arabidopsidis*, the agent that causes downy mildew in plants. These microbes perform the enhancement of plant protection better as a team than as individual players in the rhizosphere (Berendsen et al. 2018).

## Endophytes in the activation of plants’ immunity—systemic intrinsic resistance

In an effort to survive and prevail in spite of the stiff competition among microbes particularly at the rhizosphere, some microbes possessing the cellulase enzyme capable of dissolving the cellulose cell wall of plant roots gain entrance into the apoplast of the plant that includes the interior of the cell wall as well as the vascular bundle—xylem, where they live and undergo normal metabolic activities. Any microbe able to gain entrance and dwell within the plant tissue is said to be an endophyte.

These microbes also have plant growth-promoting properties such as hormone production (Naveed et al. 2015) and deaminase enzyme production, etc., for supporting the host in the fight against invading microbes. The endophytes will continue to enjoy the aid the plants render to them until the plant is mechanically uprooted or die; however, during the life of the plant, both parties benefit (Miliute et al. 2015). The presence of these organisms does not, in any way, interrupt the proper functioning of the plant and so they are not pathogenic. Other microorganisms reside on the surface of the plant root exorhizosphere microbes and still perform their duty for the interest of the plant.

Biocontrol agents provide a pathogen control role by the production of secondary metabolites that inhibit the growth of the pathogen, by out competing them or induction of systemic resistance in the plant. Endophytes as well as non-endophytic microbes can adopt either of the methods mentioned above in the control of pathogens (O’hanlon et al. 2012). Two endophytic microbes (*Diaporthe endophytica* and *Diaporthe terebinthifolii*) exerted a biocontrol effect on *Phyllosticta citricarpa* (a fungus responsible for causing citrus black spot disease in citrus fruit). These biocontrol microbes were able to inhibit the pathogen via competition and colonization of the same citrus plant organ (niche) that the pathogenic fungus will seek to colonize and cause disease in the plant (Dos Santos et al., 2016).

Endophytes found in the seed of plants are likely to enter the seed through the connection of the vascular bundle, where they will ultimately colonize the embryo and/or endosperm. They could enter the plant seed via the reproductive part of the meristems (Malfanova et al. 2013). Endophytic bacteria isolated from wheat seeds promote vegetative plant growth through phytohormone (indole acetic acid) biosynthesis, siderophore, and/or phosphate solubilization. Also observed was its ability to form biofilms. Above all, they were effective in the inhibition of the fungal pathogen *Fusarium graminearum*. These endophytes included *Paenibacillus* sp. and *Pantoea* sp. (Herrera et al. 2016). The plant growth-boosting rhizobacterium (*Paenibacillus polymyxa* AC-1) was implicated in the control/inhibition of the pathogens *P. syringae* (pv. Tomato DC 3000) and *P. syringae* (pv. *tabaci*). It was able to colonize the interior part of the *A. thaliana* plant and induce the expression of pathogenesis-related genes (PR1, PDF1.2, WRKY29, FRK1) in the plant responsible for salicylic and jasmonic acid signaling and defense pathways in *A. thaliana* (Hong et al. 2016).

In the same vein, bacteria species identified as *B. amyloliquefaciens* (SB14), *B. pumilus* (SB6), *Bacillus siamensis* (AP2), and *B. siamensis* (AP8) isolated from the rhizosphere of sugar beet and root and shoot of apple and walnut plants controlled the disease of sugar beet damping-off that is caused by *R. solani* (AG-4 and AG2-2). Among these isolates, *B*. *amyloliquefaciens* was the most effective biocontrol agent. However, solutions to every problem lie in the problem. This is supported by the observation that using native microbes associated with plants have higher chances of biocontrol success as a result of their environmental familiarity, adaptation, and easy adjustment to the host plant metabolites and the environmental conditions. This enables them to perform well in the fight against pathogens and foreign (allochthonous) microorganisms (Karimi et al. 2016).

From another perspective, the viral pathogen cucumber mosaic virus of tomato plants has been found to be controlled by *Trichoderma harzianum* through the mechanism of induced systemic resistance. *T. harzianum* primed the activation and expression of the defense genes for jasmonic acid, ethylene, and salicylic acid production in the tomato plants. It enhanced the growth of the plant, photosynthetic rate/chlorophyll content as well as the gaseous exchange capacity of the inoculated plant (Vitti et al., 2015) providing a suitable alternative to the control of viral pathogen as chemical treatment of plants is ineffective in the control of viral pathogen (Vitti et al. 2015). The saprotrophic beneficial endophytic fungus *T. harzianum* T-78 via its efficient root colonization of tomato plant not only stop the penetration/invasion and multiplication of *Meloidogyne incognita* but also primed the activation of salicylic and jasmonic acid immune dependent signaling pathways in plant. This induction of pathogenesis-related genes was in the presence of the pathogen which elicited the process by sensitizing the plant through pathogen-associated molecular pattern induction. At first, *Trichoderma* induced salicylic acid defense against the nematode and when the nematode imped the jasmonic acid signaling/expression in the plant root, the fungus quickly restored the suppressed jasmonic acid pathway and fortified the plant resistance to the nematode reproduction and proliferation (Martınez-Medina et al., 2016).

The molecular approach through which *T. harzianum* induces systemic resistance in plants involves the expression of hydrolase genes *Thph1* and *Thph2* that is controlled by Thc6 (C6 zinc finger protein). These gene products (Thph1 and Thph2) prime the production of ROS (reactive oxygen species) and increased the cytoplasmic calcium content of maize leaf. They equally increased the expression of the jasmonic acid/ethylene defense signaling pathway in the non-genetic modified maize plant for efficient protection against the disease (Saravanakumar et al., 2016). The colonization of maize plant by *T. harzianum* induced the plant’s systemic resistance to the pathogen *Curvularia lunata* by priming the expression of the gene PAF-AH (platelet activating factor acetylhydrolase). This activation factor produced by the fungus primed the expression and production of chitinase and cellulase enzyme including jasmonic acid inducible genes. The intracellular activities breed and enhance the resistance of maize to the pathogen (Yu et al. 2015).

## Elicitors in the induction of systemic resistance to biotic stress in plants

Elicitors are natural or synthesized chemicals either from microbial origin or chemical combination of elements (Table 2) that are capable of initiating systemic resistance action in plants against pathogens when applied. They could cause a physiological condition of programmed cell death/apoptosis (Heath, 1998). Apoptosis could be caused by invasive pathogen attack on plants whose influence increase the intracellular level of reactive oxygen species and initiate calcium buildup within the plant cell that results in apoptotic cell death (Li et al. 2018).

**Table 2:** The influence of biological and chemical elicitors in plant protection against pathogens

Elicitors/inducers	Plants	Phytopathogens	Priming actions of elicitor in plants	References
Azelaic acid AZA1	Arabidopsis	*Pseudomonas syringae* pv. maculicola ES4326	Defense genes enabled the movement of AZA by binding to lipid-AZA and induced systemic resistance in the plant	Cecchini et al. (2015)
Ammonium ion (NH_4_^+^)	Tomato	*Pseudomonas syringae* pv. tomato DC3000	Improved the accumulation of hydrogen peroxide which triggered the abscicis acid signaling pathway and induced the closure of stomata as well as accumulation of putrescine in the plant	Fernández-Crespo et al. (2015)
PeBA1 protein	Tobacco	*Tobacco mosaic virus*, *Botrytis cinerea*	Induced defensive genes for the production of salicylic acid, phenylalanine ammonia lyase, jasmonic acid, hydrogen peroxide, and phenolic compounds	Wang et al. (2016)
Benzothiadiazole	Tomato	Tomato spotted wilt virus and citrus exocortis viroid	Activated the salicylic acid signaling pathway and improved the plant resistance to the viral infection	Lopez-Gresa et al. (2016)
Benzothiadiazole	Sunflower	*Sclerotinia sclerotiorum*	Hindered the development of fungal hyphae in the plant and increased the establishment of mycorrhizae in the plant root	Ban et al. (2017)
Methyl jasmonate	Whitebark pine	*Cronartium ribicola* mountain pine beetle (MBP, *Dendroctonus ponderosae*)	It triggered the plant reprogramming of the transcriptome profile, a set of DEG (differentially expressed genes) associated with plant defense signaling, etc.	Liu et al. (2017)
Salicylic acid or methyl jasmonate	Cassava	*Xanthomonas axonopodis* pv*.* manihotis	Elevated the defense action of cassava plant to the bacterial pathogen	Yoodee et al. (2018)
Benzoylsalicylic acid	Tobacco, Arabidopsis	Tobacco mosaic virus	It enhanced plant resistance to the virus and induce the expression of non-expressor of pathogenesis-related gene 1 (NPR1), hypersensitivity-related molecules, mitogen activated protein kinase (MARK) as well as WRKY genes in the plant	Kamatham et al. (2016)
Ningnanmycin	Tobacco	Tobacco mosaic virus	Inhibited polymerization of tobacco mosaic virus protein coat and induced systemic resistance and accumulation of pathogenesis-related proteins in the plant	Han et al. (2014)
3-Acetonyl-3-hydroxyoxindole (AHO)	*Nicotiana tabacum*	Tomato spotted wilt virus	Induced the activation of differentially expressed genes (PR1 and PR10) that facilitated the priming and expression of metabolic pathways for synthesis of phenyl propanoid, sesquiterpenoid, triterpenoid for protecting plant cuticle, and wax	Chen et al. (2017)
N-decanoyl-homoserine lactone	Tomato	*Botrytis cinerea*	Induced plant jasmonic acid biosynthesis and signal transduction in the treated tomato plant which confer resistance to the fungal infection	Hu et al. (2018)
PevD1	*Nicotiana benthamiana*	*Verticillium dahliae*, Tobacco mosaic virus, *Pseudomonas syringae* pv*.* tabaci	Interacted with asparagine-rich protein (Nbnrp1) to regulate PevD1 that is associated with induction of cell death and increased the plant resistance to the virus	Liang et al. (2018)

Exogenous and endogenous salicylic acids are important in gene priming for systemic resistance/protection of plants against pathogens. Exogenous applied salicylic acid on tomato plant has induced the activation of genes responsible for pathogenesis and protection of plant. This substance hindered root infection by *M. incognita* and boosted the resistance of the plant to the nematode (Molinari et al. 2014).

A variety of metabolites produced by *Azospirillum brasilense* (V5 and V6) which comprises of IAA, indole-3-ethanol (IEA), indole-3-lactic acid (ILA), and SA promoted the increased expression of oxidative stress genes and pathogenesis-related genes in the leave and root parts of the maize plant. Application of the metabolites and live bacteria on the leaves of maize equally enhanced plant growth as a result of the phytohormone produced and priming of plant defense genes (Fukami et al. 2017; Vacheron et al. 2015).

Also implicated in the protection of plant against pathogen is the volatile organic compounds produced by beneficial rhizomicrobes (Table 2) which performs their role by the initiation of systemic resistance in the plants. Volatile organic compounds are gaseous, low molecular weight organic compounds such as albuterol, 1,3-propanediol (Tahir et al. 2017), 3-pentanol, and 2-butanone (Song and Ryu 2013) which can activate the plant immune system and imped pathogenic microbes from a distance. For this type of compound, distance is never a barrier to its action compared with other chemical substances of higher molecular weight like exopolysaccharide and proteins that are involved in pathogen control which acts only in close proximity/contact with the plant (Yunus et al. 2016; Xie et al. 2014; Raza et al. 2016).

Also, an important organic compound produced by the organism *Enterobacter aerogenes* was good in boosting the resistance of maize to the attacking fungus *Setosphaeria turcica* (a leaf blight-causing fungus). In as much as this organic compound (2,3 butanediol) produced by *E. aerogenes* has a remarkable effect in plant resistance to blight-causing fungus, it does not necessarily contribute to the resistance of the maize plant to parasitoid (*Cotesia marginiventris*) attack, yet when applied to the soil as an amendment act as an attractant of the microbe to the plant root. But the attraction is inhibited in the presence of the organism (*E. aerogenes*) (Table 3) (D’Alessandro et al. 2014). On the other hand, the analogue of salicylic and jasmonic acid facilitates the activation/initiation of systemic resistance in treated tobacco plant. It equally protects the plant against tobacco mosaic viral infection by priming the activation and expression of the genes responsible for plant systemic protection. Tobacco plants that bear defective salicylic and jasmonic acid genes increased the infectivity and/or susceptibility of tobacco plant to the viral infection (Zhu et al. 2014).

**Table 3:** Influence of direct microbe-plant association in plant protection

Microbes	Compounds produced in plants	Invading pathogens	Plants	References
*Azotobacter* sp., *Pseudomonas* sp.	Beta 1,3-glucanase, peroxidase	Cucumber mosaic virus	Cucumber	El-Borollosy and Oraby (2012)
*Bacillus cereus* AR156	Hydrogen peroxide, pathogenesis-related protein	*Pseudomonas syringae* pv. tomato	Arabidopsis	Niu et al. (2016)
*Pseudomonas putida* CRN-09, *Bacillus subtilis* CRN-16	Peroxidase, polyphenol oxidase, phenylalanine ammonia lyase, beta 1,3-glucanase, chitinases	*Macrophomina phaseolina*	Mung bean	Sharma et al. (2018)
*Paenibacillus* sp. P16	Induced systemic resistance in cabbage plant	*Xanthomonas campestris* pv*.* campestris	Cabbage	Ghazalibiglar et al. (2016)
*Bacillus amyloliquefaciens*	Production of peroxidase, polyphenol oxidase, and expression of pathogenesis-related genes for (jasmonic and salicylic acids)	*Ralstonia solanacearum*	Tomato	Li et al. (2017)
*Pseudomonas* sp. (BaC1-38)	Beta 1,3-glucanase, chitinases	*Xanthomonas campestris*	Rice	Lucas et al. (2014)

The volatile organic compound 2,3-butanediol has two enantiomers (2R,3R and 2S,3S) and a meso-type (2R3S) butanediol produced by root-associated beneficial microbes are implicated in the systemic resistance induction in pepper against cucumber mosaic virus (CMV) and tobacco mosaic virus (TMV). Among the three isomers, 2R, 3R and 2R, 3S, butanediols were the most effective in priming salicylic acid, jasmonic acid, and ethylene defense genes in the plant (Kong et al. 2018).

However, the application of Beta aminobutyric acid (BABA) (a non-protein amino acid elicitor) and non-host *Phytophthora nicotianae* on chili peppers induced systemic resistance of the plants to the pathogen *Phytophthora capsici*. These elicitors influenced the plant by reducing plant cellular sucrose concentration as well as tricarboxylic acid cycle intermediates. It also boosted the concentration of hexose phosphate, hexose disaccharides, amino acids, and galactose in the induced plant, thereby building the plant immunity against *Phytophthora capsici* (Stamler et al. 2015).

The dead cells surrounding the area of infection will block the migration of the pathogen from point of infection to other points in the plant. This is to encourage the production of antimicrobial substances that will impede the proliferation of the pathogen (Hammerschmidt, 1999). Exogenous application of synthetic salicylic acid and beta aminobutyric acid (in a concentration of 1.5 and 15 mM, respectively) was found to induce the activation of the pathogenesis-related protein production of chitinase enzyme as well as beta 1,3-glucanase for effective immunity and resistance of tomato plant to the invasive pathogen *Alternaria solani* (Raut and Borkar, 2014). Also, hydrogen peroxide, abscisic acid, and 2,4 dichlorophenoxy acetic acid chemical inducers applied exogenously to potato plant challenged with *A. solani* was able to resist the pathogen infection as a result of increase in plant intracellular concentration of peroxidase, phenylalanine ammonia lyase, and polyphenoloxidase enzymes. These synthesized enzymes inhibited the invasion of *A. solani* in tomato plant (Nassar and Adss 2016).

Calcium treatment of plants is another abiotic approach to enhancing plant resistance to biotic stress. Calcium has been found to boost the activities of peroxidase and strengthen the plant cell wall as well as improve the production of substance that could inhibit fungi development on plant (Clark 2013; Xu et al. 2013; Downie, 2014). A combined treatment of tomato plant with calcium salt and salicylic acid elevated the production of antioxidant proteins, chitinase, and pathogenesis-related proteins that encouraged tomato resistance to *B. cinerea* infection (Linlin et al. 2016).

Another elicitor that is eco-friendly and effective in induction of plant resistance to pathogens include plant extract. Plant extract—limonoids (Munronin O)—obtained from the plant of *Munronia henryi* Harms is effective in protecting tobacco plants against the tobacco mosaic virus by enhancing the defense enzyme production and salicylic acid level of the treated tobacco plant and induced systemic acquired resistance in the plant (Yan et al. 2018). And the interaction of these chemical with soil humus or particles leave a big doubt concerning their possibility of being biodegradable when they form stable complexes with the soil particles. Therefore, caution must be applied in the use of these chemical analogues to boost plant immunity. However, natural elicitors such as plant extracts and microbial metabolites use should be encouraged. But the challenge remains that the cost of producing these natural organic elicitors as well as their preservation could be quite expensive.

## Conclusion

The use of pesticides to control plant pathogens and pests cause issues of concern as the majority of the agrochemicals used in biocontrol not only lower the disease severity in the plant but also lower the yield of the crop (Egel et al. 2018). Some of these chemicals can be harmful to human and animals and may constitute environmental pollution. Carbamate and pyrethoid (insecticides) can cause secondary outbreaks of pests such as aphids (Egel et al. 2018). This necessitates the search for a suitable and eco-friendly alternative in disease control and management.

The use of microbes capable of antagonistic behavior against pathogens for induction of systemic resistance in plant is a good method in crop disease management (Babalola 2010). Also, the application of elicitors either in a drench form or foliar spray on plants is yet another method of pathogen control. Elicitors are capable of inducing the expression and/or activation of pathogenesis-related-genes and improving the immunity of the treated plant for efficient fight against invaders.

However, to achieve a maximum protection of plant against pathogens, an integrated disease management and control approach that will involve the use of microbes, its metabolites, synthetic chemicals, and plant extracts formulation that will be simultaneously applied to the plant will enable farmers win the war against plant pathogens, increase crop yield, and achieve a sustainable agricultural practice in ensuring food security.
